# Co-assortment in integron-associated gene cassette assemblages in environmental DNA samples

**DOI:** 10.1186/1471-2156-11-75

**Published:** 2010-08-10

**Authors:** Carolyn A Michael, Nigel R Andrew

**Affiliations:** 1 Department of Biology, Macquarie University, Sydney, NSW, Australia; 2 Centre for Behavioural and Physiological Ecology, Zoology, University of New England, Armidale, NSW, Australia

## Abstract

**Background:**

It has been shown that integron-associated gene cassettes exist largely in tandem arrays of variable size, ranging from antibiotic resistance arrays of three to five cassettes up to arrays of more than 100 cassettes associated with the vibrios. Further, the ecology of the integron/gene cassette system has been investigated by showing that very many different cassettes are present in even small environmental samples. In this study, we seek to extend the ecological perspective on the integron/gene cassette system by investigating the way in which this diverse cassette metagenome is apportioned amongst prokaryote lineages in a natural environment.

**Results:**

We used a combination of PCR-based techniques applied to environmental DNA samples and ecological analytical techniques to establish co-assortment within cassette populations, then establishing the relationship between this co-assortment and genomic structures. We then assessed the distribution of gene cassettes within the environment and found that the majority of gene cassettes existed in large co-assorting groups.

**Conclusions:**

Our results suggested that the gene cassette diversity of a relatively pristine sampling environment was structured into co-assorting groups, predominantly containing large numbers of cassettes per group. These co-assorting groups consisted of different gene cassettes in stoichiometric relationship. Conservatively, we then attributed co-assorting cassettes to the gene cassette complements of single prokaryote lineages and by implication, to large integron-associated arrays. The prevalence of large arrays in the environment raises new questions about the assembly, maintenance and utility of large cassette arrays in prokaryote populations.

## Background

Integrons include a site-specific genetic system capable of mobilising and rearranging genes packaged as mobile gene cassettes [1]. In concert with other DNA mechanisms capable of mobilisation between cells, the integron/gene cassette system contributes to the overall process of lateral gene transfer (LGT). LGT is a major contributor to genetic diversity amongst prokaryotes [2] and hence a significant force in prokaryote evolution. The ability of the integron/gene cassette system in particular to influence the evolution of prokaryote strains is graphically shown in the rapid dissemination of antibiotic resistance genes both geographically and amongst different prokaryotes [3]. However, cassette-associated genes are not limited to the provision of antibiotic resistance. While the majority of the ORFs (open reading frames or putative genes) contained within gene cassettes have no analogue in sequencing databases, those few for which a function has been attributed, have been adaptive in nature [4,5].

An integron generally consists of an integrase gene, *IntI*, its promoter *P_Int_*, an attachment site *attI*, which is recognised by the IntI integrase, and a promoter *P_c_*. Gene cassettes, the class of DNA elements mobilised by an integron, typically consist of an ORF, and an attachment site termed *attC *(or *59-be *elsewhere), which also provides a recognition site for the IntI integrase. The integron mobilises DNA by inserting or excising gene cassettes through the action of the IntI integrase at sites defined by both *attI *and *attC*. Repeated rounds of insertion result in the formation of a tandem array of gene cassettes adjacent to the integron, commencing at the *attI *site [6]. Because *P_c _*is oriented towards the cassette array through *attI*, those cassette-associated genes proximal to *P_c _*may also be expressed [7].

A number of gene cassette arrays have been observed in detail to date [8-10]. These arrays can contain in excess of 200 gene cassettes in strains of the vibrios. Interestingly, phylogenetic examination of closely related strains containing large arrays such as the *Vibrio *pandemic strains, has showed multiple cassette indels between otherwise identical cassette arrays [11] suggesting that the dynamics of gene cassettes within an array may not be limited to single cassette mobilisation. However, an examination of the dynamics of gene cassettes within the wider prokaryote communities has not been explored. While cassette diversity in environmental samples is substantial [12-14], relationships amongst different types of gene cassette have yet to be addressed in an environmental context. An understanding of environmental interactions among gene cassettes is of fundamental importance because patterns of co-assortment in the environment may firstly reflect the linkage seen amongst gene cassettes within arrays, as well as offering the ability to monitor changes in the constitution of arrays *in situ*. Consequently, in this work we asked the following questions:

1) Do gene cassettes co-assort in the environment?

2) To what extent does any co-assortment represent the presence of gene cassette arrays or other genetic linkage between cassettes?

3) Are large gene cassette arrays, as seen in sequencing studies, a common vehicle for containing gene cassettes in the environment?

4) What patterns of co-assortment are present in a natural environment?

## Methods

### Sampling

Spatially explicit soil samples were taken from a dry sclerophyll woodland in North Ryde, Australia, (151.07°079'E. 34..46°108'S WGS1984). The soil is derived from Hawkesbury Sandstone, is well-drained and low in nutrients, particularly P and N. The environment at the time had been experiencing a period of relatively high temperatures and protracted drought. Two parallel 5 m transects, 10 m apart were used. Six 5 ml soil samples were taken at 0, 0.01, 0.1, 0.5, 1.0 and 5.0 m distances from an origin point along each transect. Samples were stored overnight at 4°C prior to environmental DNA extraction and subsequent analysis.

Single soil samples were taken from additional sites at ranges up to 200 km. These sites included a pristine site at Hornsby rifle range (151.05°.079'E 33.41°.179'S WGS 1984).

### Environmental DNA extraction

The extraction of representative DNA from environmental soil samples followed the methods of Yeates *et al. *[15]. Replicate DNA extractions were performed on all soil samples.

### Cassette size class screening (CSCS) PCR

Cassette size class screening PCR follows the method of cassette PCR as outlined in Stokes *et al *2001 [16] in order to visualise amplicons using a DNA sequencer, PCR primers described in the cassette PCR method included a 5' 6-FAM fluorescent tag. Triplicate cassette size class screening PCRs were performed on two samples in transect 1 to establish reproducibility.

### PCR of co-assorting cassettes

Per reaction 50 μl: 2.0 μl x sample DNA, 50 nM MgCl, 10 nM dNTP, 1.0 μl × 1 mg/ml Rnase, 50 pM cassette-specific fwd primer, HS527 50 pM cassette-specific rev primer, HS528 0.3 μl × Red Hot Taq and 5.0 μl × 10× PCR buffer (Abgene, Surrey, UK). *Thermal profile: *80°C hot start, 94°C for 10 minutes initial denaturation then 35 cycles of 94°C/30 sec, 60°C/30 sec, 72°C/1 min 30 sec. Followed by a terminal 72°C for 10 minutes. Primers are listed in Table 1.

**Table 1:** PCR Primers

PCR PRIMER	SEQUENCE 5' > 3'	COMMENTS
HS527/HS286	6-FAM, GGGATCCTC(S)GCT(K)GARCGA(M)TTGTTAG(V)C	attC fwd
HS528/HS287	6-FAM, GGGATCCGC(S)GCT(K)ANCTC(V)(R)(R)CGTTAG(S)C	attC rev
MRG 53b	GATGGCGTCGCGAGTCAACC	258bp fwd
MRG 54	GCCTTGCAATCCGTCAGCCACG	258bp rev
MRG 55	AGTCAGACCTCAGGAACAAGAGC	347 bp fwd
MRG 56	CAGCCTTTGCTCTTACTGCGAACC	347 bp rev
MRG57a	CTGCTTCCATTGTAAGAACACC	429 bp fwd
MRG58a	TCGGCACAAGTACAGTCTATGC	429 bp rev
MRG59a	TCTACGATCGCTATGGCAACGA	482 bp fwd
MRG60a	TGAGCCAATATTGAGGCAAGCA	482 bp rev
16SF	AGAGTTTGATCMTTGGCTCAG	16S fwd
16SR	TACGGYTACCTTGTTACGACTT	16S rev

### Quantitative PCR (QPCR)

For Quantitative PCR, we used Roche Light Cycler™instrumentation (Roche Applied Sciences, Mannheim, Germany)

10 μl reactions were run using 50pM of each primer (Table 1), 1.0 μl x sample DNA, 40 nM MgCl, 10 pM primer and 1.0 μl of SYBR Green™master mix (Roche Applied Science, Mannheim Germany) per reaction. Primers used are MRG 53b/MRG 54 for the 258 bp cassette, MRG 55/MRG 56 for the 347 bp cassette, MRG 57a/MRG 58a for the 429 bp cassette and MRG 59a/MRG 60a for the 482 bp cassette. Primers are detailed in Table 1. The temperature profile included a 10-minute initial Taq activation step at 95°C per the manufacturer's recommendations, 35 cycles of 94°C/15 seconds, 60°C/15 seconds and 72°C/30 seconds. Final cool down at 20°C for 10 minutes. PCR products were run on a 2% TAE gel to verify the presence of amplicons of expected size and the absence of non-specific amplifications.

### Long-Range PCR

Long-range PCR used the following primer combinations. For the 258/347 bp gene cassette pair, primers MRG 53b/54, MRG 55/56, MRG 53b/56, MRG 55/54, MRG 53b/55, MRG 54/56 were used. For the 429/482 bp gene cassette pair primers MRG 57a/58a, MRG 59a/60a, MRG 57a/60a, MRG 59a/58a, MRG 57a/59a, MRG 58a/60a were used (Table 1).

Per 50 μl reaction: 1.0 μl × sample DNA, 5.0 nM dNTP, 50 nM MgSO_4_, 2.5 μl × Invitrogen Platinum 10× master mix, 0.2 μl × Invitrogen Platinum Taq. 50 pM cassette specific fwd primer, 50 pM cassette specific rev primer. *Thermal program: *94°C/30 seconds initial denaturation, 35 cycles of 94°C/20 seconds, 65°C/25 seconds, 68°C/20 minutes. Final hold at 4°C.

### 16S TRFLP

Per 50 ul reaction: 2.0 μl × sample DNA, 50 nM MgCl, 10 nM dNTP, 1.0 μl × 1 mg/ml Rnase, 50 pM 16S fwd primer, 50 pM 16S rev primer, 0.3 μl.× Red Hot Taq (Abgene, Surrey, UK), 5.0 μl × 10 × PCR buffer (Abgene, Surrey, UK). *Thermal profile: *80°C hot start, 94°C for 10 minutes initial denaturation then 35 cycles of 94°C/30 sec, 60°C/30 sec, 72°C/1 min 30 sec. Followed by 72°C for 10 minutes.

Aliquots of the fluorescent PCR products were digested separately with two different restriction endonucleases, *Rsa *1 and *Hin*f 1 according to manufacturer's protocols (Promega, Wisconsin, USA). The 16S rRNA TRFLP digestion products and CSCS PCR amplicons were analysed using the Genescan protocol of the ABI 377 DNA sequencer.

### High-resolution electrophoresis

The fluorescent products of the CSCS PCR were analysed on an ABI 377 DNA sequencer by supplying 1 ul aliquots of each PCR reaction, using denaturing gels at approximately 2500V for 1.5 hours. Polyacrylamide gels (6%) were poured with the addition of TEMED and ammonium persulphate after degassing to enhance polymerisation. 1500 bp size standards from ABI were used preferentially for increased low-end resolution and due to the absence in the samples taken of significant fragments larger than 1000 bp. The system was run typically at 2500V for approximately 4 hours to achieve single base pair resolution over the range 20-1500 base pairs. Peaks below 70 base pairs in size were excluded as being primers or primer artifacts. Likewise, peaks below a fluorescence value of 20 units were excluded due to the low Signal/Noise value.

### Distribution analysis

The data set from the CSCS PCR included correlated peak height and size class information and was consolidated in a spreadsheet. Size classes were rounded to unit base pair and then applied to a 1-1000 bp size continuum for each sample and the results of duplicate DNA extractions averaged. Size classes less than 70 bp were discarded as primer dimer artifacts and PCR noise. Subsequently, remaining peaks in the replicate DNA extractions, PCRs and digests were averaged across replicates and the percentage standard error calculated. Thereafter, mean peak height values for each size class in each soil sample were assembled into a further spreadsheet with samples ordered along the collection transects.

### Sorting populations by spatial distribution

To determine if different cassette size classes co-assorted, each gene cassette size class within the spreadsheet-based data distribution, was firstly scored for the number of DNA samples in which it appeared. Additionally, each cassette size class was scored on its presence in either or both sampling transects. The distributions were then rearranged so that gene cassette size classes sharing the same spatial distribution were adjacent in the table (Additional file 1).

### Measuring co-assortment

Relationships amongst cassettes between samples along both transects was assessed using non-metric Multidimensional Scaling (nMDS) in Primer 6.1 [17]. Amplicon abundance data was log(x+1) transformed to improve the underlying distribution of the data.

### Null Modelling

The spatial distribution of gene cassette size classes was assessed using null models [18], to assess the degree to which the spatial distributions might arise from chance alone. Null models used in this analysis determined the degree of randomness within a distribution [19] with respect to the two parameters of interest in this study: size class and sample site. Therefore, null modelling returned a probability that an arrangement of gene cassette size classes sharing a particular spatial distribution within the data set was due to chance alone. Null models were performed on the gene cassettes size class data using EcoSim 7^® ^[20]. A co-occurrence model, the C-score index [21], was used to test for species co-occurrence in a presence/absence matrix. Five thousand iterations of a random assemblage matrix were compared using a random seed number to begin the sequence. Row sums were fixed and column sums were equi-probable. The average amount of co-occurrence among all unique pairs of species in the assemblage is measured by the C-Score. This measure is superior to other indices in both Type I and Type II error properties [20]. We used one simulation algorithm which is considered robust and recommended by Gotelli & Entsminger [20]: Sim9: Fixed rows-fixed columns. This simulation maintains fixed row and column sums. It has a good Type I error rate, and is powerful at detecting patterns in noisy data sets when used with the C-score.

**Table 2:** ECOSIM calculated results, Gene Casettes

Observed index	2.17
Mean of simulated indices	1.86
Variance of simulated indices	0.00036
p(observed > = expected)	< 0.00001
Standardized Effect Size	16.88

## Results and discussion

The environmental DNA samples used in this work had previously been examined for gene cassette diversity [12]. In that work, it was also established that the amplicons returned by the fluorescent cassette (CSCS) PCR, represented identifiable gene cassette sequences. Consequently, the amplicons returned from the CSCS PCR were termed gene cassette size classes.

**Figure 1:**
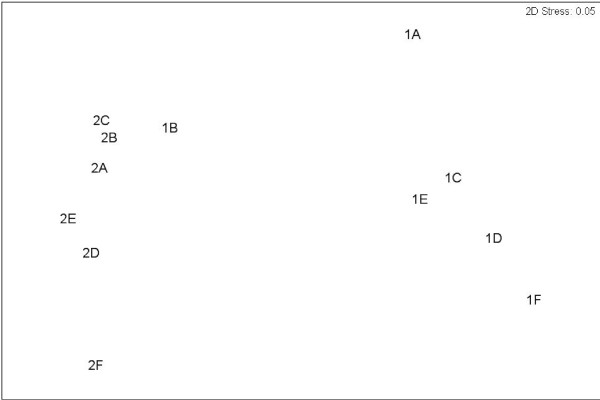
**Non-metric Multidimensional Scaling (nMDS) plot of the Gene Cassette assemblage collected from five distances (0, 0.01, 0.1, 0.5, 1, 5 m labelled A-F inclusive) along the two transects (1 and 2) 10 metres apart**.

Initial analysis of the gene cassette size class data set showed that cassette size classes did not assort randomly (Table 2, p observed > = expected: < 0.0001). This was seen most clearly in the non-metric multidimensional scaling plot (Figure 1), where the degree of similarity of cassette populations between different sample sites mirrored their physical locations, thus showing relationship amongst sample sites. In order to further investigate this non-random arrangement of cassette size classes, a more detailed examination of the distribution was undertaken:

Only two of the cassette size classes were detectable in all environmental DNA samples. The remainder were detectable only in subsets of the DNA samples. It was noted that for the majority of the cassette size classes, their distribution within the sampling area was contiguous. That is, the presence of a gene cassette size class within one DNA sample generally implied its presence in adjacent samples in the same transect.

The majority of detected cassette size classes co-assorted with other cassette size classes within the sampling area. That is, the particular spatial distribution of a gene cassette size class amongst the environmental DNA samples was shared by other gene cassette size classes, so forming a "co-assorting group'. The most obvious type of co-assorting group was where a number of cassette size classes were present only in a single DNA sample. An example of such a group was the 16 different cassette size classes that occurred only in the D sample of transect 2 (Additional file 1). A further type of co-assorting group consisted of a number of different cassette size classes present in multiple DNA samples. An example of this type of group was the 18 different cassette size classes that only occurred in transect 2, samples E and F (Additional file 1).

**Figure 2:**
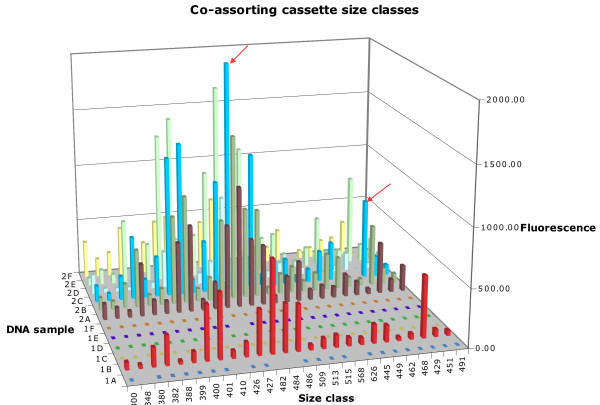
**A subset of a large co-assorting group (Groups A and B, additional file **1) **seen in multiple DNA samples**. Co-assorting groups share similar peak intensity distributions between environmental DNA samples. For example, the arrowed peaks representing 429 bp and 482 bp size classes retain their relative heights in all samples in which they are present.

Amongst co-assorting groups containing many cassette size classes, across multiple environmental DNA samples, it was noted that the size class with the largest peak height in one DNA samples was generally the size class with the largest peak height in the other DNA samples in which the group was present. This relationship also held amongst other member peak heights excepting where the peaks were very large or small and so approached the performance limits of the instrumentation used. This meant that the "fingerprint' of the relative peak heights of the different size classes of a group in one DNA sample was also evident in other environmental DNA samples. For example, a group containing 18 different size classes across seven of the environmental DNA samples, labelled "Group A' in Additional file 1, is shown in Figure 2. The same pattern of peak height conservation appeared in some groups of size classes that occupied a subset of the DNA samples of a more widespread group. An example of this is seen in Group B in Additional file 1, where cassette size classes were present in all except one of the environmental DNA samples in which Group A was present (Figure 2).

Co-assorting groups with greater than four different cassette size class members involved more than 80% of the 585 different gene cassette size classes detected. Similarly, 20 distinct co-assorting groups, each containing greater than ten different size classes could be distinguished when the cassette data set was sorted for spatial distribution.

### Do co-assorting groups of cassette size classes reflect genetic linkage?

Where a cell line contains an example of the integron/gene cassette system, cassette arrays containing many different members are often present. Gene cassettes within such arrays, being on the same DNA molecule, are in stoichiometric relationship. Consequently, we hypothesised that the co-assorting cassette size classes observed in this work represented multiple gene cassettes from one or more arrays within a single bacterial lineage. We examined this hypothesis by testing for stoichiometric relationship between cassette size classes within a co-assorting group.

Co-assorting groups containing many cassette size classes across multiple environmental DNA samples were chosen for this work. This choice allowed a comparison of the relative amounts of two representative cassettes in different locations, while still providing a negative control in those DNA samples where the co-assorting group did not occur. Representative cassette size classes that differed significantly in size (bp) were selected to obviate possible PCR artefacts such as poly-A tailing.

**Figure 3:**
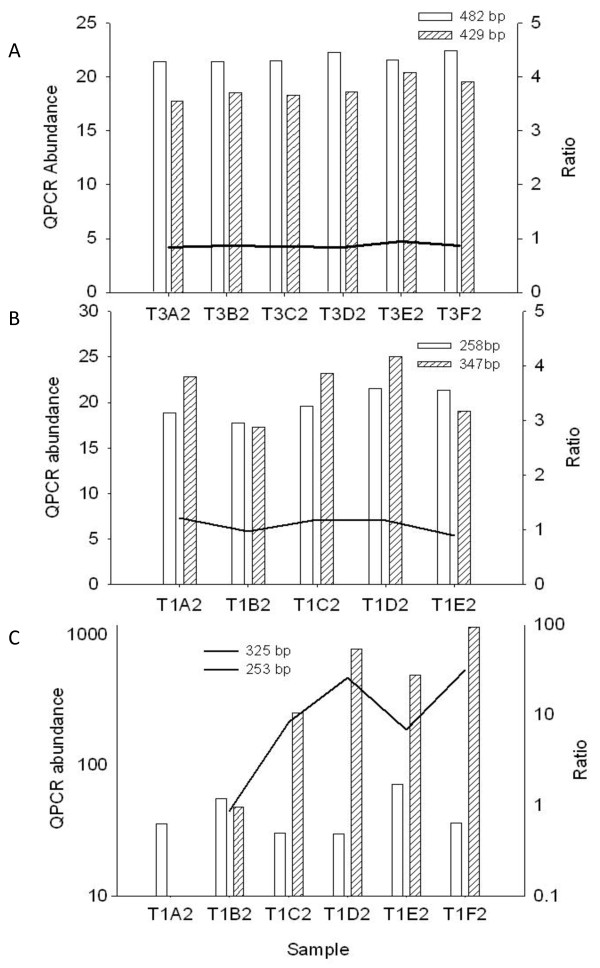
**Quantitation of potentially linked gene cassettes using QPCR**. In this series of panels, the left hand vertical axis represents abundance as measured by QPCR. The horizontal axis indicates the different environmental DNA samples in which the abundance of the target gene cassettes was measured. The right hand vertical axis shows the ratio of the abundances of the two gene cassettes considered, demonstrating a stoichiometric relationship through a constant ratio between DNA samples. Graph A indicates the 429/482 bp co-assorting pair, Graph B indicates the 258/347 bp co-assorting pair and Graph C shows the 253/325 bp non co-assorting pair as discussed in the text.

Group A-B, (Additional file 1 and Figure 2) was selected for this comparison study. The 482 bp cassette size class from group A, the largest peak height size class in this group was selected for examination. Selection of a 429 bp size class as the second size class from the analogous Group B (Additional file 1, Figure 2) provided a stringent test of the proposed stoichiometric relationship. The relative abundances of the 482/429 bp cassette pair were in constant ratio where they occurred in the sampling area demonstrating that these two cassettes were in stoichiometric relationship (Figure 3A). Similarly, a 258 bp and 347 bp gene cassette selected from a different co-assorting group (Additional file 1) were compared and shown to be in stoichiometric relationship (Figure 3B). Finally, a third pair of gene cassettes, a 253 bp cassette and a 325 bp cassette, that were not members of the same co-assorting group, was examined as a control (Figure 3C). This 253/325 bp pair did not maintain the same relative abundance across environmental DNA samples in which they both occurred, indicating that these non-co-assorting cassettes were not in a stoichiometric relationship.

Sequences of the 482 bp and 429 bp gene cassettes were found to be novel and are shown in Additional files 2 and 3, while the 258 bp and 347 bp gene cassettes were determined to be Bal 29 (Genbank AF349051) and Bal 28 (Genbank AF349084) cassettes respectively [22]. All sequenced cassettes contained ORFs, none of which showed significant matches with the existing genomic databases beyond homology with previously sequenced gene cassettes. PCRs using the specific primers derived from these cassettes were used to probe the suite of environmental DNA samples taken from the sampling area. The presence of appropriately sized amplicons in only those samples containing the original size class verified that targeted cassettes were members of the appropriate co-assorting groups.

The PCR methodology used in this work does not recover all gene cassettes within a DNA sample due to diversity amongst *attC *sequences in gene cassette populations. Consequently, the number of gene cassette size classes detected under-samples the true diversity present in the sample. However, the methodology used produces amplicons, identifiable as gene cassettes, with a specificity of greater than 95% [16] and the cassettes amplified are not restricted to individual cassette arrays. Consequently, the subset of the environmental cassette assemblage returned by this method was not substantially biased. The detection method used, being based on amplicon sizing, also does not discriminate between different cassettes of the same size [12] and so further under-samples the environmental gene cassette diversity. Therefore, the gene cassette size class data set used in this work was a representative rather than comprehensive estimate of the gene cassette metagenome in this environment. Nevertheless, the stoichiometric relationship uncovered between the gene cassettes that were found to comprise the co-assorting groups of cassette size classes indicated their location within a copy-number-controlled environment. While a copy-number-controlled environment could be construed to be cassettes within the same integron-associated array, the prevalence of prokaryote lineages containing multiple integrons [23], suggests that the co-assorting groups could equally indicate cassettes within the same cell but present in different integron associated arrays. So, encompassing both of these possibilities, we considered that a single co-assorting group as found here, represented a subset of the gene cassette complement of an individual prokaryote lineage within the sampling environment.

### Assessing linkage between co-assorting gene cassettes

In order to examine the possibility that gene cassettes in co-assorting groups might be part of the same array, PCR primers used to identify co-assorting gene cassette pairs were also used in long range PCRs on the environmental DNA samples. By using alternate primers specific to each gene cassette, four possible orientations between the two target cassettes were examined to determine any linkage between the cassettes of a co-assorting group, as well as to examine any intervening DNA.

The 258/347 bp gene cassette pair produced a 3.1 Kbp amplicon from long-range PCR. This product was seen in only one of the environmental DNA samples containing these cassettes. This PCR used a forward primer specific for the 258 bp gene cassette (MRG 53b) and a reverse primer for the 347 bp gene cassette (MRG 56) indicating that these two cassettes were in the same orientation, as defined by the orientation of their respective *attC *sites. Further, this orientation was the same as typically seen amongst the *attC *sites of cassettes within arrays. PCRs on the 3.1 Kbp amplicon, using both 258 bp (MRG 53b/MRG 54) and 347 bp specific primer pairs (MRG 55/MRG 56) showed that complete copies of both gene cassettes were present on the 3.1 Kbp fragment. A final PCR of the 3.1 Kbp fragment using generic *attC *site primers (HS 286/HS 287), showed the presence of intervening gene cassettes between the 258 bp and 347 bp gene cassettes, further indicating the presence of these two cassettes within an array.

The 429/482 bp cassette pair produced no reproducible amplicon in any long-range PCR in any of the environmental DNA samples. It is noteworthy that the 258/347 bp gene cassettes originated from a co-assorting group containing only eight cassette size classes. Additionally, these eight size classes differed in length from the 258 bp and 347 bp cassettes by no more than six bp and so probably represented PCR artefacts in the amplification of a short array. In contrast, the 429 bp and 482 bp cassettes originated in a group containing more than 20 distinctly different size classes. Consequently, it was more likely that the 258/347 bp pair were closely linked within an array than the 429 bp and 482 bp cassettes. Therefore, the likelihood of successful amplification of the 429/482 bp pair if contained within a large array was necessarily reduced.

### Do the same cassettes co-assort in other environments?

Gene cassettes are mobile genetic elements and so the cassette complement of a prokaryote lineage might be expected to vary under the continued influence of LGT. The analytical method used in this work to establish the presence of co-assorting groups could not be used to detect changes in the cassette complements of individual lineages within the sampling area, as changes in the constitution of a cassette group across the sampling area would produce two distinct co-assorting groups. However, the PCR primers defining individual cassette pairs within co-assorting groups, as developed to determine stoichiometric relationships, could also be used to assess other environmental DNA samples for the presence of these target cassettes.

Amongst a selection of 22 environmental DNA samples taken from a diverse range of environments within Australia, the 429/482 bp pair of cassettes was found in no other environment. However, both members of the 258/347 bp pair were found in one of the alternate environments (Hornsby rifle range), approximately 20 km from the site used for the majority of this study. Additionally, this pair of cassettes was previously identified in soils taken from the Balmain power station site and identified as Bal29 and Bal28 respectively. The Balmain site was approximately 15 km distant from the sample area used in this study. In contrast to the other sites in which the 258/347 bp pair was found, the Balmain site was characterised by heavy metal and hydrocarbon contamination. The observation that these two cassettes retained their association over large geographical distances and in disparate environments suggested that the linkage between these cassettes was robust. Consequently, it was inferred that cassette mobilisation may not necessarily be a common event in these environments. This corroborated observations of the significant numbers of inactivated integrons seen in environmental sequencing studies [23].

### The distribution of gene cassettes in the sampling area

The detection of co-assorting cassettes and their identification as cassette complements of individual prokaryote lineages allowed conclusions to be drawn regarding the arrangements of gene cassettes in prokaryote populations:

There were at least 2043 different gene cassettes within the sampling area [12]. In this study, we considered a representative sample of this gene cassette assemblage, being the 585 distinct size classes returned by the analytical methods used. Of these 585 size classes, more than 70% could be ascribed to 20 discernable co-assorting groups containing more than ten cassette size classes. As shown in this work, these co-assorting groups were representative of the gene cassette complements of distinct prokaryote lineages present in this environment. From this perspective, the following observations were made regarding the gene cassette assemblage in the sampled environment.

Firstly, lineages with large complements of gene cassettes, the largest being 46 different size classes in this study, contained the majority of the gene cassette diversity in this environment. Secondly, the distribution of individual co-assorting groups did not generally extend across the sampling area but rather exhibited "patchy" but spatially contiguous distributions within the sample area. For example, the group from which the 258 bp and 347 bp cassettes were taken was present in samples A through E in transect 1 but not in sample F which was more than three metres away from its neighbour, sample E. Neither did this group appear in any of the transect 2 samples.

Thirdly, it had been previously established that approximately 10% of prokaryote phylotypes contain one or more integrons [23,24]. In the environmental DNA samples used in this work, it was found from paired 16S TRFLP analyses using different restriction enzymes, which both returned essentially the same number of 16S species, that there were approximately 504 distinct prokaryote phylotypes. Consequently, we predicted that approximately 50 of the phylotypes present would contain integrons and consequently, gene cassettes. Therefore, the 20 prokaryote cassette complements containing ten or more size classes, together with the at least 20 groups containing less than ten size classes detected in this work, represented a close approximation of the expected number of cassette-bearing phylotypes. However, it has also been shown that the number of cassette size classes detected in this sampling area significantly underestimated the local cassette diversity [12]. In this environment, therefore, the expected number of integron-containing lineages had been approximated by the number of observed co-assorting groups, while the cassette diversity remained a significant underestimate. While there are a number of possible explanations to account for this discrepancy, it was most likely that the additional gene cassette species were undetected members of the co-assorting groups present. If this were the case, the array or arrays [23] associated with each cassette-bearing lineage would generally be large. Consequently, the relatively small arrays seen in association with the highly mobile, antibiotic-resistance bearing, class 1 integrons would be the exception rather than the rule in this environment and possibly in the wider cassette metagenome.

This last observation calls into question the adaptive utility of the integron/gene cassette system. It is currently thought that gene cassettes proximal to the integron are preferentially expressed [25]. Accordingly, for gene cassettes to reliably provide a selective advantage to the host through their expression, an array must necessarily be short: typically of the order of 8-10 gene cassettes to ensure reliable expression as has been the case in the well studied class 1 integron [7]. It has been hypothesised that large cassette arrays such as those seen in *Vibrio spp*. express only those cassettes proximal to the integron with the distal cassettes forming a cassette library to be accessed through rearrangements of the array [25]. In this work, the preponderance on large cassette complements in the environment, coupled with the apparent stability of at least some cassette associations, suggested that the large cassette complements and hence arrays represented a current adaptive advantage rather than a readily accessible adaptive "library' [25] or long-term replicative burden to the host lineage. The means by which large arrays might provide such an adaptive advantage could be explained by the existence of intra-array promoters and consequent expression of distal cassettes: a theory in need of further assessment.

### Further applications of the measurement technique used in this work

A study of the reproducibility of the normalised peak heights of individual cassette size classes from replicate DNA extractions and PCRs showed a high reproducibility for peak heights greater than 20 and less than 2000 fluorescence units, with a SE% (percentage standard error) value of less than 5%. Parallel comparison of the normalised peak heights for the same cassette size class in different DNA samples containing the same co-assorting group, showed more variability with an average SE% of 15%. It was assumed that this variability was due to differing quantities of constituent gene cassettes in each size class apart from the cassette that was part of the co-assorting group.

The reproducibility of relative peak heights for a co-assorting group independent of DNA sample suggested that a combination of the cassette size classes and their relative peak heights of their amplicons are useful "fingerprints' of the many cassette complements in this environment. These "fingerprints' may be used to monitor changes in the cassette assemblages, both spatially and temporally, by detecting changes in either or both the characteristic cassette size classes present or in the normalised peak heights. Lateral gene transfer in natural prokaryote populations can then be examined systematically.

## Conclusions

We have found that the gene cassette diversity of a relatively pristine sampling environment is predominantly structured into a number of co-assorting groups of cassettes. These co-assorting groups consist of gene cassettes in stoichiometric relationships. Conservatively, we have then attributed co-assorting cassettes to the gene cassette complements of single prokaryote lineages.

Amongst integron-bearing prokaryote lineages, complements containing large numbers of different cassettes were found to be relatively common in the sampling environment. As a result, we expect that large gene cassette complements and hence large integron-associated gene cassette arrays are not uncommon and may predominate within environmental gene cassette assemblages.

## Authors' contributions

CM conceived the work, executed the experiments and co-wrote the publication. NA performed the data reductions, statistical analyses and co-wrote the publication.

Both authors have read and approved the final manuscript.

## Supplementary Material

Additional file 1 **Data set**. Gene cassette size class data greater than 70 bp in size and greater than 20 Fluorescence units in intensity. The numbers within the table indicate the fluorescence intensity of individual amplicons and are the average of two replicate DNA extractions from each sample site. The data has been scored (occurrence columns) and sorted for distribution. Rows highlighted in blue denote size classes discussed in the text. Click here for file

Additional file 2 **482 bp co-assorting gene cassette alignment**. Sequences recovered using 482 bp cassette specific primers MRG 59a and MRG 60a from sample site T2F are compared to the originally recovered 482 bp (482/12) sequence. Shading indicates areas of conserved sequence within the alignment. Click here for file

Additional file 3 **429 bp co-assorting gene cassette alignment**. Sequences recovered using 429 bp cassette specific primers MRG 57a and MRG 58a, from samples sites T2B and T2F are compared to the originally recovered 429 bp sequence (429/29). Shading indicates areas of conserved sequence within the alignment. Click here for file
