# Supplementary figures and images for: Co-assortment in integron-associated gene cassette assemblages in environmental DNA samples
Source: BMC Genet. 2010 Aug 10;11:75. doi: 10.1186/1471-2156-11-75 (PMC2927473; doi:10.1186/1471-2156-11-75)

## Slide 1
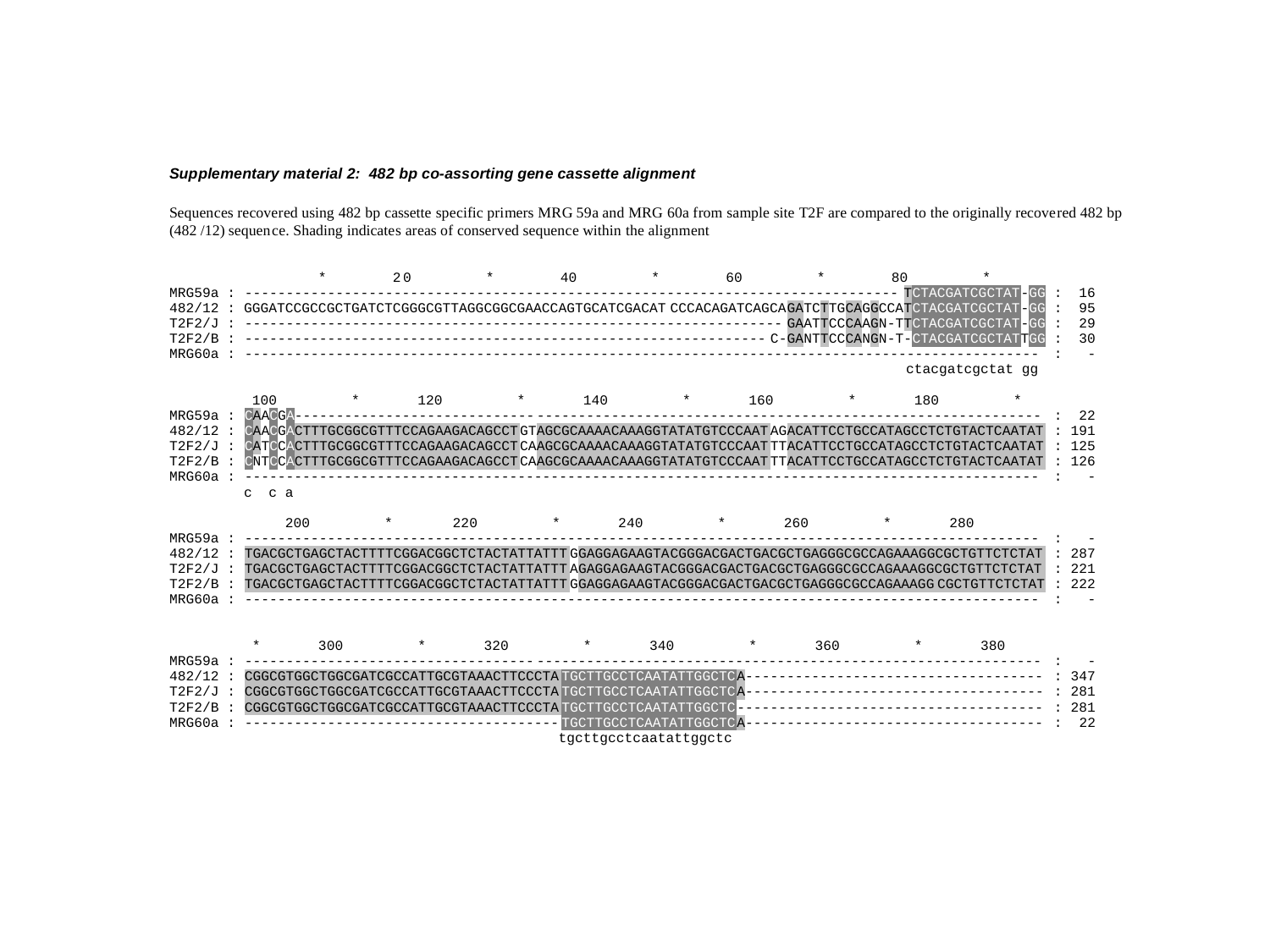

Supplement: Additional file 2 — 482 bp co-assorting gene cassette alignment. Sequences recovered using 482 bp cassette specific primers MRG 59a and MRG 60a from sample site T2F are compared to the originally recovered 482 bp (482/12) sequence. Shading indicates areas of conserved sequence within the alignment. [file 1471-2156-11-75-S2.PPT]

## Slide 1
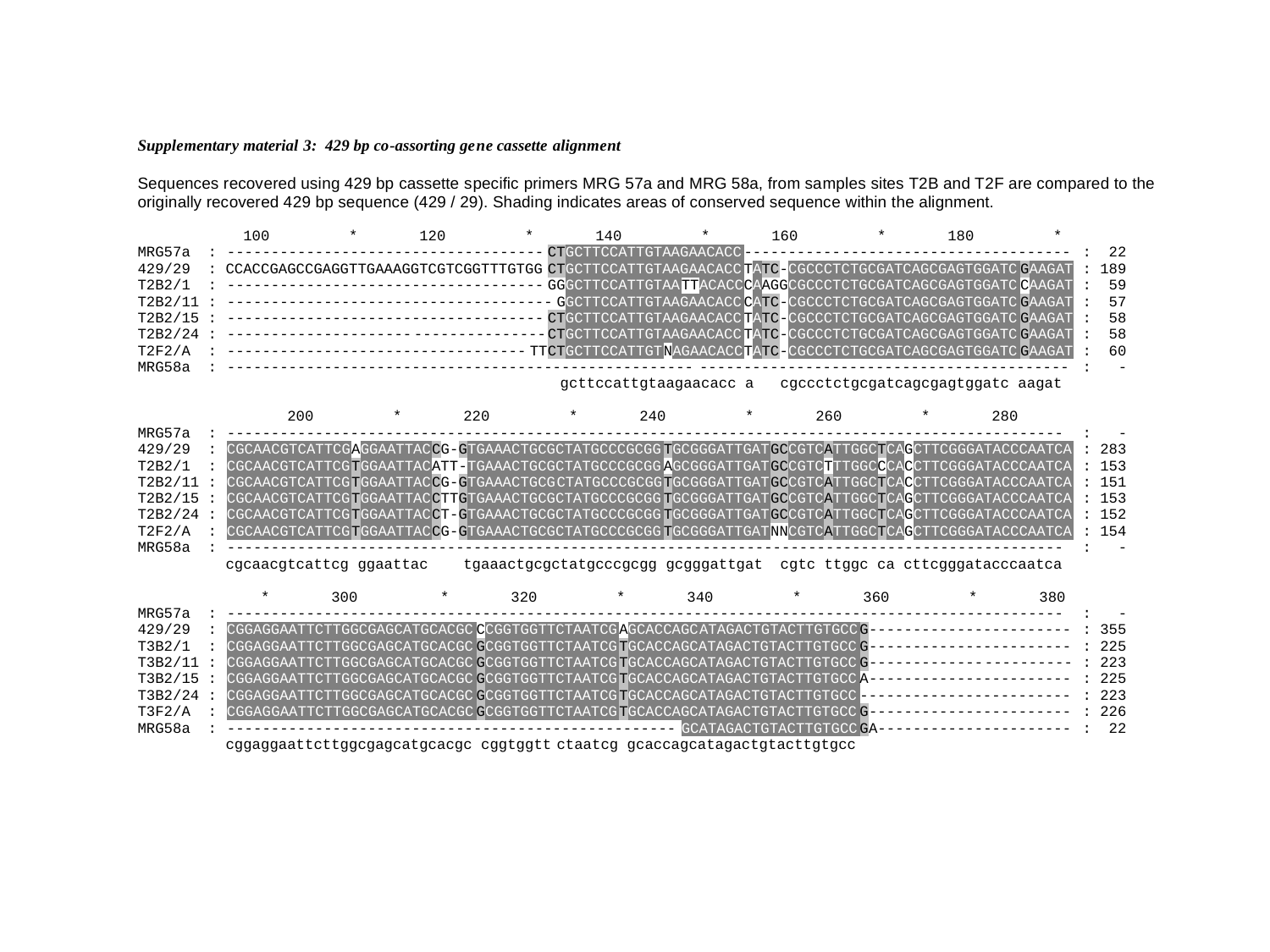

Supplement: Additional file 3 — 429 bp co-assorting gene cassette alignment. Sequences recovered using 429 bp cassette specific primers MRG 57a and MRG 58a, from samples sites T2B and T2F are compared to the originally recovered 429 bp sequence (429/29). Shading indicates areas of conserved sequence within the alignment. [file 1471-2156-11-75-S3.PPT]
